# Pea Proteins Have Anabolic Effects Comparable to Milk Proteins on Whole Body Protein Retention and Muscle Protein Metabolism in Old Rats

**DOI:** 10.3390/nu13124234

**Published:** 2021-11-25

**Authors:** Jérôme Salles, Christelle Guillet, Olivier Le Bacquer, Carmen Malnero-Fernandez, Christophe Giraudet, Véronique Patrac, Alexandre Berry, Philippe Denis, Corinne Pouyet, Marine Gueugneau, Yves Boirie, Heidi Jacobs, Stéphane Walrand

**Affiliations:** 1 Unité de Nutrition Humaine (UNH), Université Clermont Auvergne, INRAE, CRNH Auvergne, 63000 Clermont-Ferrand, France; jerome.salles@inrae.fr (J.S.); christelle.guillet@uca.fr (C.G.); olivier.lebacquer@inrae.fr (O.L.B.); christophe.giraudet@inrae.fr (C.G.); veronique.patrac@inrae.fr (V.P.); alexandre.berry@inrae.fr (A.B.); philippe.denis.2@inrae.fr (P.D.); corinne.pouyet@inrae.fr (C.P.); marine.gueugneau@inrae.fr (M.G.); yves.boirie@inrae.fr (Y.B.); 2 Cosucra-Groupe S.A., 7740 Warcoing, Belgium; cmalnero-fernandez@cosucra.com (C.M.-F.); hjacobs@cosucra.com (H.J.); 3 Service de Nutrition Clinique, CHU Clermont-Ferrand, 63000 Clermont-Ferrand, France

**Keywords:** pea proteins, plant proteins, sarcopenia, skeletal muscle, protein digestibility, muscle protein metabolism

## Abstract

Plant proteins are attracting rising interest due to their pro-health benefits and environmental sustainability. However, little is known about the nutritional value of pea proteins when consumed by older people. Herein, we evaluated the digestibility and nutritional efficiency of pea proteins compared to casein and whey proteins in old rats. Thirty 20-month-old male Wistar rats were assigned to an isoproteic and isocaloric diet containing either casein (CAS), soluble milk protein (WHEY) or Pisane™ pea protein isolate for 16 weeks. The three proteins had a similar effect on nitrogen balance, true digestibility and net protein utilization in old rats, which means that different protein sources did not alter body composition, tissue weight, skeletal muscle protein synthesis or degradation. Muscle mitochondrial activity, inflammation status and insulin resistance were similar between the three groups. In conclusion, old rats used pea protein with the same efficiency as casein or whey proteins, due to its high digestibility and amino acid composition. Using these plant-based proteins could help older people diversify their protein sources and more easily achieve nutritional intake recommendations.

## 1. Introduction

Alongside animal proteins, plant proteins are a critical part of the equation to help meet future protein demand and achieve worldwide food security. In the US, demand for plant proteins grew by 20% in both 2018 and 2019 [1]. This growing interest in plant proteins is driven by multiple factors, such as food safety concerns, rising food intolerances, increased accessibility of vegetarian and vegan foods, environmental concerns, sustainability imperatives, and consumer adoption of proactive approaches to health and wellbeing. The nutritional benefits of these new protein sources are still under investigation, with studies looking into their health benefits while also exploring their limits, such as allergenicity or anti-nutritional substance content [2]. Consumer acceptability needs to be carefully defined, as it remains the final bottleneck for developing new protein sources.

Grain legumes are a valuable source of plant food proteins, and so rising protein demand is expected to increase the dietary importance of grain legumes. Pulses generally have a higher nutritional value than other crops, especially since the onset of domestication and genetic selection processes operated by humans. Pea proteins have enough essential amino acid (EAA) content (30%) to meet WHO/FAO/UNU-recommended requirements [3]. Note that EAA requirement is based on a recommended adult protein intake of 0.8 g/kg body weight/day. Note also that peas provide well above the recommended leucine requirements [4]. In addition to providing proteins with suitable EAA profiles, legumes contain digestible carbohydrates, and some of them also contain fat.

There is considerable interest in the potential of using plant-based proteins to support muscle mass maintenance and/or growth, as demonstrated by the number of recent papers studying the impact of intakes of plant-based protein, e.g., pea proteins, on skeletal muscle anabolic response in athletes [2,5]., Dairy whey protein is a shared choice for protein supplementation in athletes because of its leucine content, its digestibility, and its ability to activate muscle protein synthesis. Most extant research on plant proteins in athletes has set out to compare and evaluate the effects of dietary supplementations with whey and pea proteins in conjunction with resistance training on muscle anabolism and strength. Taken together, the data revealed that whey and pea protein treatments led to similar responses to resistance exercise. Whey and pea proteins promote comparable muscle strength, physical performance, and body composition following resistance training [6], especially in beginners or people returning to weight training [7].

These same plant proteins could be equally valuable in other populations, such as older people, to help maintain muscle mass and slow down the aging-related process of sarcopenia. However, despite their reported efficacy in athletes, the effects of pea and other plant proteins in older people suffering from sarcopenia have not yet been disclosed. The fact that pea protein provides well above the recommended leucine requirements points to it playing a potentially valuable role in combating the loss of skeletal muscle mass and function in older subjects. Leucine is an anabolic amino acid with proven effectiveness for the maintenance of muscle mass during aging [8]. Meeting the body’s quantitative daily demand for EAA is vitally important; the quality of protein consumed by older people is an equally important factor, and is generally determined by its digestibility and utilizability by the body. Among milk proteins, whey protein digests quickly, while casein digests slowly as it clots at acidic pH in the stomach. Numerous experiments have set out to determine whether fast or slow digestion was better for muscle protein synthesis and muscle building. The bottom line is that rapid digestion is best for stimulating muscle protein synthesis and increasing muscle mass, even in older people [9]. Interestingly, a previous study has shown that pea protein transiently aggregates in the stomach and has an intermediately-fast intestinal bioavailability midway between those of whey and casein [10].

When new sources of dietary proteins are tested for nutritional quality, the first studies are carried out using animal models, as advised by FAO. The second step in such studies is often to evaluate the interest of the protein in some pathophysiological situations characterized by a reduced capacity to assimilate and metabolize proteins, as is the case in older subjects. These animal studies make it possible to precisely assess protein metabolism in certain key tissues such as skeletal muscle. Such a study is difficult to perform in humans. For pea proteins, although its digestibility is high in young rats, there is little data on the nutritional value of pea proteins in old rats as compared to dairy proteins, and particularly in terms of protein digestibility and metabolism. To address this gap, this study used old rats to evaluate the efficiency of pea proteins as compared to dairy proteins, i.e., casein and whey proteins, in terms of protein digestibility, body protein retention, muscle protein synthesis and degradation and muscle protein accretion.

## 2. Materials and Methods

### 2.1. Animal Experiment

All animal procedures were approved by the local institutional animal care and use committee (Comité d’Ethique en Matière d’Expérimentation Animale Auvergne: C2EA-02) and conducted in accordance with the European guidelines for the care and use of laboratory animals (2010-63UE) (Authorization number: APAFIS#5329-2016051115541284 v2). Animals were housed in the INRAE’s Human Nutrition Research animal facility (Agreement No. D6334515).

A total of thirty 20-month-old male Wistar rats were obtained from Janvier Labs (Le Genest-St-Isle, France). All animals came from the same batch and were bred under the same conditions throughout their lives. The rats were housed in individual cages under controlled environment conditions (12-h light/12-h dark cycle, temperature 22 °C) with free access to water. All of the rats were fed a maintenance diet (A04, Safe, Augy, France) ad libitum for a 2-week acclimatization period. Rats were then randomized into three groups according to body weight, fat mass and lean mass. Animals were assigned (*n* = 10 per group) to a diet containing either 14% casein (Armorprotéines, Saint-Brice-en-Cogles, France) (CAS rats), 14% soluble milk protein, i.e., Protarmor™ 80, a Whey protein concentrate (Armorprotéines, Saint-Brice-en-Cogles, France) (WHEY rats) or 14% pea proteins, i.e., Pisane™ (Cosucra, Warcoing, Belgium) (PEA rats) for 16 weeks. The three experimental diets were isoproteic and isocaloric (Table 1 and Table 2). Different protein to nitrogen conversion factors were used depending on the protein source used. Specifically, the conversion factors used were: 6.15 for casein, 6.08 for whey and 5.36 for pea protein. Dietary AA levels were analyzed by the ABioC laboratory (Arzacq, France) according to EN ISO 13903:2005 standard method (Table 1). Body weight and food intake were measured weekly. At the end of the experiment and after an overnight fast, the remaining CAS (*n* = 6), WHEY (*n* = 6) and PEA (*n* = 8) rats were anesthetized. Blood samples were collected from the abdominal aorta and drawn into precooled ethylenediaminetetraacetic acid (EDTA) tubes. After centrifugation, plasma was removed and frozen at −80 °C until analysis. Liver, heart, adipose tissues and hindlimb skeletal muscles were weighed, snap-frozen in liquid nitrogen, and stored at −80 °C for later analysis.

### 2.2. Whole Body Composition

At the beginning, middle (after 8 weeks) and end (after 16 weeks) of the experiment, fat and lean body mass (g) were measured in non-anesthetized living animals placed in an EchoMRI-100 body composition analyzer (Echo Medical Systems LLC, Houston, TX, USA).

### 2.3. Protein Quality Evaluation

To collect total urine and feces, rats were placed in metabolic cages (Tecniplast France, Decines-Charpieu, France) for 4 days in the last week of the experimental protocol. Total excreted nitrogen was then determined by the Dumas method at Institut UniLaSalle (Beauvais, France) [11]. Dietary protein quality was evaluated by calculating nitrogen balance (NB), apparent protein digestibility (AD), true protein digestibility (TD), net protein utilization (NPU) and biological value (BV) using the following equations [12]:

NB(g)=NI−(FN+UN)

$$\mathrm{AD}\;\left(\%\right)=\frac{\mathrm{NI}-\mathrm{FN}}{\mathrm{NI}}\times 100\;$$

$$\mathrm{TD}\;\left(\%\right)=\frac{\mathrm{NI}-\left(\mathrm{FN}-\mathrm{EFN}\right)}{\mathrm{NI}}\times 100$$

$$\mathrm{NPU}\;\left(\%\right)=\frac{\mathrm{NI}-\left(\mathrm{FN}+\mathrm{UN}-\mathrm{EFN}-\mathrm{EUN}\right)}{\mathrm{NI}}\times 100$$

$$\mathrm{BV}\;\left(\%\right)=\frac{\mathrm{NPU}}{\mathrm{TD}}\times 100\;$$

where NI is nitrogen intake, FN is fecal nitrogen, UN is urinary nitrogen, EFN is endogenous fecal nitrogen, and EUN is endogenous urinary nitrogen. A group of old rats that received a nitrogen-free diet during the metabolic cage period was used to deduce fecal and urinary endogenous nitrogen excretions.

### 2.4. Plasma Analyses

Plasma levels of fasting glucose, triglycerides, and total cholesterol were determined using a Konelab 20 analyzer (Thermo-Electron Corporation, Waltham, MA, USA). ELISA kits were used to determine insulin (Alpco Diagnostics, Salem, NH, USA), leptin, (Biovendor, Bmo, Czech Republic), adiponectin (AssayPro, St Charles, MO, USA), TNFα (Millipore, Molsheim, France) and IL-10 (Diaclone, Besançon, France). Homeostatic model assessment of insulin resistance (HOMA-IR) was calculated to assess insulin sensitivity in old rats, using the formula:

$$\mathrm{HOMA}-\mathrm{IR}=\frac{\left(\mathrm{fasting}\;\mathrm{glucose}\times \mathrm{fasting}\;\mathrm{insulin}\right)\;}{22.5}\;$$

with fasting glucose level expressed as mmol/L and fasting insulin level expressed as mIU/L.

### 2.5. Protein Synthesis Measurement

To study muscle protein synthesis, we measured rate of incorporation of a stable isotope, i.e., an AA L-[^13^C_6_]-labeled phenylalanine (Eurisotop Saint-Aubin, France), into muscle proteins using the flooding dose method. Fasting rats were injected subcutaneously with a large dose of L-[^13^C_6_] phenylalanine (50% mol excess, 150 µmol/100 g) to flood the precursor pool of protein synthesis. Incorporation time of labeled phenylalanine was 50 min. A 50-mg piece of plantaris muscle was used to isolate and hydrolyze total mixed proteins as previously described [13]. After derivatization, L-[^13^C_6_] phenylalanine enrichments in hydrolyzed proteins and in tissue fluid were assessed using gas chromatography–mass spectrometry (Hewlett-Packard 5971A; Hewlett-Packard Co., Palo Alto, CA, USA). Fractional synthesis rates (FSR) of proteins were calculated using the equation:

(1) $$\mathrm{FSR}=\frac{\mathrm{Ei}}{\mathrm{Ep}\;\times t}\times 100$$

where Ei is enrichment as atom percent excess of L-[^13^C_6_] phenylalanine derived from phenylalanine from proteins at time t (minus basal enrichment), Ep is mean enrichment in the precursor pool (tissue fluid L-[^13^C_6_] phenylalanine), and t is incorporation time in hours.

### 2.6. Western-Blot Analysis

Homogenates of frozen plantaris muscles were prepared as previously described [14]. Denatured proteins were separated on a polyacrylamide gel and electrotransferred to a polyvinylidene difluoride membrane (Millipore, Molsheim, France). After blocking with 5% skimmed dry milk in Tris-buffered saline (TBS) + 0.1% Tween-20, membranes were incubated with primary antibodies: p70 S6 kinase (Thr389) and anti-total p70 S6 kinase (Cell Signaling Technology, Ozyme distributor, Saint-Quentin-en-Yvelines, France). After washing with TBS + 0.1% Tween-20, immunoblots were exposed to swine anti-rabbit immunoglobulins conjugated with horseradish peroxidase (HRP) (DAKO, Trappes, France). The antigen/primary antibody/secondary antibody/HRP complexes were visualized by luminescence using ECL Western Blotting Substrate (Pierce, Thermo Fisher Scientific, Courtaboeuf, France) and a Fusion Fx imaging system (Vilber Lourmat, Collegien, France). Quantification of band density was done using MultiGauge 3.2 software (Fujifilm Corporation, Tokyo, Japan). The values represented the ratio of the phosphorylated protein levels to total protein levels, and were expressed in arbitrary units.

### 2.7. mRNA Analysis

The protocol for total RNA extraction and mRNA analysis has been previously described [14]. Briefly, a piece of plantaris muscle was homogenized in Tri-Reagent (Euromedex, Mundolsheim, France) and total RNA was isolated according to manufacturer’s instructions. RNA amount was measured by spectrophotometry at 260 nm. Total RNA was reverse-transcribed using SuperScript III reverse transcriptase and a random hexamer and oligo dT primer combination (Invitrogen, Life Technologies, Saint-Aubin, France). PCR amplification was performed using a Rotor-Gene Q system and 2 × Rotor-Gene SYBR Green PCR master mix (Qiagen, Courtaboeuf, France). Relative concentrations of mRNA corresponding to genes of interest were quantified using Rotor-Gene software and the standard curve method. The primers used for real-time PCR analysis were listed in Table 3. Hypoxanthine-guanine phosphoribosyltransferase (HPRT) was used as housekeeping gene. Data were expressed in arbitrary units.

### 2.8. Mitochondrial Enzymatic Assays

First, 50 mg of frozen rat plantaris muscle was homogenized in homogenization buffer (225 mM mannitol, 75 mM sucrose, 10 mM Tris-HCl, 10 mM EDTA, pH 7.2) and then centrifugated at 650× *g* for 20 min at 4 °C. The supernatant was kept and the pellet was suspended in homogenization buffer and resubmitted to the same procedure. Both supernatants were pooled and used for activity measurements [14,15,16]. Complex I and 3-hydroxyacyl-CoA dehydrogenase (HAD) activities were spectrophotometrically assayed in the supernatant fraction by following the oxidation of nicotinamide adenine dinucleotide, reduced (NADH). Citrate synthase (CS) activity was measured by following the reduction of 5,5-dithiobis (2-nitrobenzoic acid) (DTNB) [14,15,16,17]. Activities were expressed in nmol/min/mg of proteins.

### 2.9. Statistics

To calculate the sample size, we used published and unpublished data of net protein utilization (NPU) [18]. A difference of 20–25% and a mean variance of 10% were expected for this parameter between CAS group and WHEY group. Based on these data, the setting of type I error (α) at 5% and a power of 90%, a total of 6 rats per group was required. To anticipate potential rat death for the 16-week experimental period, 10 rats were assigned to each diet. All results were presented as means ± SEM. Animals that died or developed tumors during the experiment were excluded from the analysis. In detail, while we had 10 rats per group at baseline, the number of rats remaining at the end of the experiment was 6 CAS rats, 6 WHEY rats, and 8 PEA rats. The data were analyzed for homogeneity of variance and normality. Homogeneous data were analyzed by a one-way analysis of variance (ANOVA) followed by a Tukey-Kramer test to evaluate the significance of inter-group differences. Heterogeneous data were analyzed using Kruskal-Wallis test and the significance of inter-group differences was assessed using a Steel–Dwass test. Differences were considered significant at *p* < 0.05. Statistical analysis was performed using NCSS 2020 software (NCSS LLC., Kaysville, UT, USA).

## 3. Results

### 3.1. Caloric Intake, Body Composition Evolution, and Final Tissue Weights

No significant difference in calculated daily caloric intake was observed between experimental groups throughout the study period (86.0 ± 4.6 kcal/day, 92.0 ± 3.2 kcal/day and 94.8 ± 5.9 kcal/day for CAS, WHEY and PEA rats, respectively). Rat groups were purpose-defined at the beginning of the experiment to ensure no significant between-group differences in body weight, fat mass and lean mass. Thereafter, body weight, fat mass and lean mass remained not significantly different between CAS, WHEY and PEA rats at each timepoint (i.e., the middle (week 8) and the end (week 16) of the experiment) (Table 4). In accordance with the body composition measurements, the weights of several lean tissues, (i.e., skeletal muscle, liver and heart) and two different fat tissues (i.e., perirenal adipose tissue and subcutaneous adipose tissue) presented no significant between-group differences at the end of the experiment (Table 5).

### 3.2. Protein Quality Evaluation

Nitrogen intake and fecal and urinary nitrogen contents were evaluated during the metabolic cage period (Table 6). None of these parameters were significantly different between rat groups. Nitrogen balance, which is the difference between nitrogen intake and nitrogen loss by both fecal and urinary routes, was similar between CAS, WHEY and PEA rats (Table 6). There were no significant between-group differences in apparent digestibility, which considers all of the digestive processes involving protein digestion, including endogenous nitrogen losses, or in true digestibility, which considers the specific digestion of dietary protein by subtracting endogenous nitrogen losses. Finally, net protein utilization, which is the ratio of retained nitrogen to ingested nitrogen, and biological value, which is the ratio of retained nitrogen to absorbed nitrogen, were similar between CAS, WHEY and PEA rats (Table 6).

### 3.3. Plasma Metabolic Parameters and Cytokines

Fasting levels of lipid metabolic markers, i.e., triglycerides and total cholesterol, were not significantly different between CAS, WHEY and PEA rats (Table 7). There were no significant dietary source-protein effects on parameters related to insulin sensitivity, i.e., fasting glucose and insulin concentrations and calculated HOMA-IR. Circulating leptin concentrations were similar between experimental groups, while adiponectin levels tended to be higher in PEA rats compared to CAS rats and WHEY rats (*p* = 0.07). After 16 weeks of feeding with dietary treatment, rats showed similar plasma concentrations of pro-inflammatory cytokines such as IL-1β and TNFα, and the anti-inflammatory cytokine IL-10 (Table 7). To evaluate inflammatory status, we calculated the ratios of the inflammatory markers TNF-α and IL-1β to the anti-inflammatory marker IL-10. TNFα/IL-10 and IL-1β/IL-10 ratios did not differ between groups (Table 7).

### 3.4. Markers of Muscle Protein Anabolism and Catabolism

**Figure 1:**
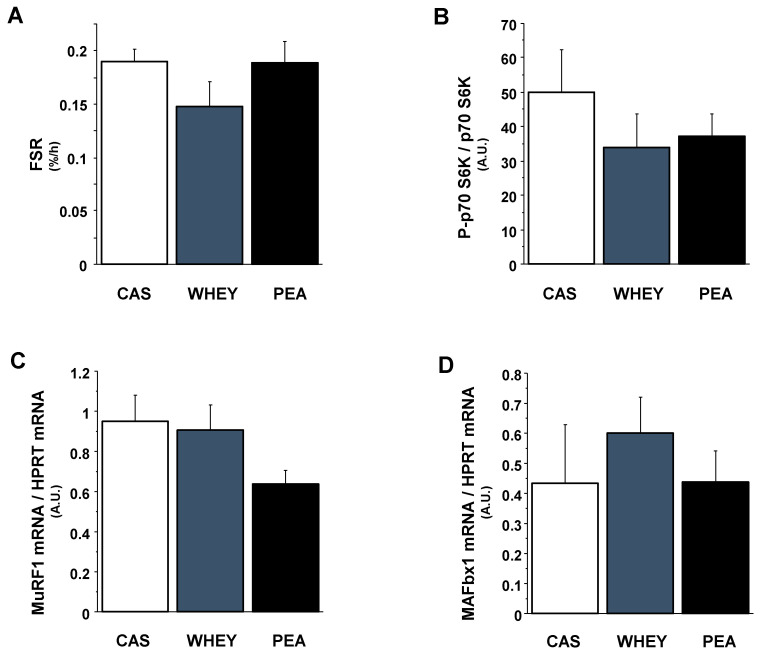
Effects of different experimental diets on protein synthesis and expression of ubiquitin-proteasome pathway markers in plantaris muscles of old rats. Fractional synthesis rate (**A**) was measured by tracer enrichment in plantaris muscles after a 50-min incubation with L-[^13^C_6_] phenylalanine. In the same muscles, the phosphorylation states of p70 S6 kinase (**B**) were determined by Western-blotting, and the gene expressions of the two ubiquitin E3 ligases MuRF1 (**C**) and MAFbx (**D**) were analyzed by quantitative RT-PCR analysis. Statistical significance was assessed by ANOVA, followed by a Tukey-Kramer test or a Kruskal-Wallis test followed by a Steel–Dwass test depending on homogeneity of variance and normality. Data are expressed as means ± SEM. A.U.: Arbitrary units.

Fractional synthesis rates (FSR) were measured in plantaris muscles of old rats (Figure 1A). According to skeletal muscle mass measurements, muscle FSR was similar between CAS, WHEY and PEA rats. Associated with these data, protein quality did not affect the phosphorylation rates of p70 S6 kinase (an intermediate of the translation initiation step) in plantaris muscles of old rats (Figure 1B). The involvement of the ubiquitin-proteasome pathway in the regulation of skeletal muscle mass in the three experimental groups was assessed by measuring mRNA expressions of MuRF1 and MAFbx. Gene expressions of both E3 ubiquitin ligases were also unchanged by experimental diets in rat skeletal muscles (Figure 1C,D).

### 3.5. Muscle Mitochondrial Activity

**Figure 2:**
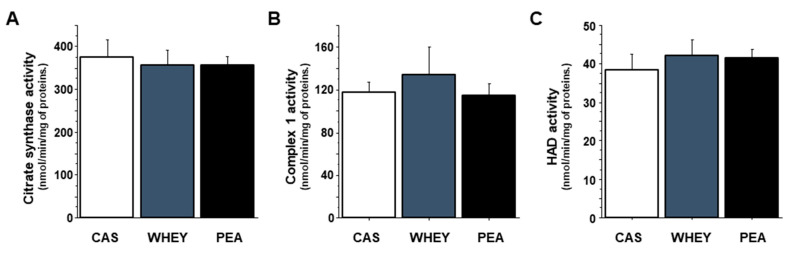
Mitochondrial enzyme activity in skeletal muscles of old rats after 16 weeks of different diets. Mitochondrial function was assessed by measuring citrate synthase (**A**), complex 1 (**B**) and 3-hydroxyacyl-CoA dehydrogenase (**C**) activities in plantaris muscles. Statistical significance was assessed by ANOVA, followed by a Tukey-Kramer test or a Kruskal-Wallis test followed by a Steel–Dwass test, depending on homogeneity of variance and normality. Data are expressed as means ± SEM.

To explore the effect of protein quality on muscle mitochondrial function in old rats, we measured the maximal activity of citrate synthase, which is a mitochondrial matrix enzyme often used as a marker of mitochondrial density. CAS, WHET and PEA rats showed similar citrate synthase activities in plantaris muscles (Figure 2A). Likewise, the activities of muscle complex 1 and 3-hydroxyacyl-CoA dehydrogenase (HAD), i.e., one of the electron transport chain complexes and a key enzyme of the mitochondrial β-oxidation cycle, respectively, were not affected by the different experimental diets (Figure 2B,C).

## 4. Discussion

Protein quality is an important component of protein intake to support growth, development, and maintenance of essential body tissues and functions [19]. The nutritional value of a protein depends on how its AA balance matches to needs, in particular EAA, and on its digestibility, i.e., on the release of AA and small peptides ready for intestinal absorption [20]. Proteins from alternative sources, such as plant proteins, are often described as having less balanced EAA profiles and lower digestibility than animal-sourced proteins [21]. However, there is a lack of data directly comparing the nutritional values of animal and plant proteins under the same experimental conditions, especially in older subjects. Here, we examined the effects of a 16-week pea protein diet on protein digestibility, body weight and composition, tissue weight, metabolic indexes, and muscle protein turnover and metabolism in old rats. Pea protein was compared to two dairy proteins, i.e., whey protein and casein, that are considered to be among the best-quality proteins, especially for maintaining body composition and muscle mass and function during aging [22]. Overall, we clearly showed that in old rats, a 16-week ingestion of milk proteins or pea protein did not influence protein assimilation and nitrogen retention, particularly in skeletal muscle. It should therefore be possible to use such plant-based protein sources for older people, which would make it possible to diversify intake and more easily attain the nutritional recommendations for this population.

### 4.1. Nitrogen Balance, Digestibility and Rate of Utilization

When studies set out to compare the nutritional quality of several dietary proteins, the first issue to consider is usually how effectively the proteins are assimilated by the body. In particular, it is important to measure nitrogen balance, digestibility and rate of utilization to get a picture of the capacity of the protein to get digested and absorbed and to get assimilated in the tissues. Overall, the data on nitrogen balance, true digestibility and net protein utilization showed that the three proteins tested in this work had a similar effect in old rats. First, the apparent and true digestibilities of pea proteins were in the same range of values of the other proteins. Recent studies have reported that pea protein is highly digestible in rats [18,23]. However, this work represents one of the first studies to show that pea protein is also highly digestible in old rats. It has been suggested that the digestibility of plant proteins is impaired due to the presence of both anti-nutritional factors and indigestible fractions in their sequence [23]. However, the pea protein used here was a protein isolate, and protein isolates are generally well-digested [24]. In addition, protein isolates are particularly low in anti-nutritional factors, due to the manufacturing process used to extract the protein [25]. High protein digestibility induces a high quantity of AA available for intestinal absorption and, thus, improves the nutritional value of the protein source [26]. Hence, net protein utilization was equivalent between old rats fed pea protein, casein or whey protein. Urinary and fecal nitrogen excretion in old rats did not differ between the three groups, leading to an equivalent whole-body nitrogen retention. This observation contrasts with other studies done in pigs that reported increased urinary nitrogen excretion and plasma urea levels in response to soybean protein compared to casein [27]. We previously showed in young rats that protein utilization increased after feeding animals with wheat pasta enriched with fava bean flour as compared to an isoproteic wheat pasta enriched with gluten. However, in this work, protein utilization still remained lower than that measured in rats fed casein [28]. However, when the same study was carried out in old rats, there was no difference between the group fed wheat pasta enriched with fava bean and the group fed casein [18].

Evaluation of the nutritional quality of dietary proteins relies not only on protein digestibility but also on its AA composition, notably its EAA content. The EAA composition of the pea protein used in this study was close to casein and to the needs of rats, according to National Research Council [29]. The AA composition of pea protein is characterized by a limiting content of methionine (Met) [30], but the total sulfur AA content is adequate [29]. Consequently, the net protein utilization and biological value measured in old rats were equivalent regardless of the protein used in the diet. Note that this result could be explained not only by EAA composition, in particular a high leucine content, but also by the high digestibility of the pea protein. To sum up, we showed that the biological value of ingested nitrogen, in particular nitrogen retention, did not differ in old rats, regardless of whether the protein in the diet was casein, whey, or pea protein.

### 4.2. Body Composition and Skeletal Muscle Mass

In the present study, although we observed an age-related physiological trend towards increased body fat and reduced lean mass between the first and last month of the study, the protein source in the diet did not significantly change body composition in old rats. This result was also confirmed by the tissue weights at the end of the 16-week period. In accordance with the whole-body composition measurements, the weight of tissues constituting the lean mass, i.e., skeletal muscles, liver and heart, and of tissues resulting from the fat mass did not differ between different dietary protein groups. Few studies have focused on comparing the effects of animal versus plant proteins on body composition in old rats. We previously evaluated (also in old rats) the nutritional value of pasta made from a mix of wheat semolina and legume flours, i.e., fava bean, lentil, or pea flour [18]. Two groups were fed diets with casein or whey protein as protein source, and three groups were fed diets made with fava bean pasta, lentil pasta or pea pasta as protein source. The study found that body weight and composition, i.e., fat mass and lean mass were not significantly different between groups at each timepoint, i.e., the beginning, the middle, and the end of the experiment [18]. The effect of dietary protein sources on body composition and tissue weight has been evaluated in other works, but these studies were generally done in young rats. A lower lean mass gain was observed in young rats given soy protein for 28 days than in young rats fed whey protein [31]. At the muscular level, other studies found that, compared to casein, 16 to 20 days of ad libitum consumption of proteins from legumes, i.e., beans or lentils provoked lower muscle weights in young rats [32,33,34]. In addition, Alonso et al. found that muscle mass and muscle protein content were lower in young rats fed seed peas than in young rats receiving casein. In this latter study, peas were extruded and cooked to reduce the antinutritional factor content [35]. The change in lean mass or skeletal muscle mass after long-term consumption of plant-based meals has not been thoroughly assessed in older people. The rare studies available have shown that the consumption of plant proteins, when provided at sufficient amounts in each meal (i.e., >30 g/meal), should be able to maintain lean and muscle mass, and therefore increase the potential to mitigate sarcopenia in older subjects [5,36,37]. Taken together, the data presented here showed that some plant proteins, e.g., pea proteins, promoted a similar effect on body composition and muscle mass to casein and even whey protein in old rats, and could therefore be tested in the elderly as an intervention to counteract sarcopenia.

### 4.3. Mechanisms

Several mechanisms may explain the similar action of milk proteins and pea protein on body composition and muscle mass in old rats. First, analysis of the AA content of each protein showed equivalent leucine contents between pea protein and casein. There is clear evidence that during aging, the leucine content of dietary proteins is an important parameter impacting its anabolic effect on lean mass, and specifically skeletal muscle mass [38]. It is now well recognized that leucine acts as an anabolic signal by stimulating protein synthesis and inhibiting protein breakdown at muscle level. For instance, leucine supplementation for 10 days attenuated the decrease in expression of eukaryotic translation initiation factors in young and old rat muscles [39]. In addition, this supplementation decreased the levels of ubiquitinated proteins and inhibited proteasome activity in old rats [40]. The leucine content of pea protein could thus explain its effectiveness on muscle protein turnover and therefore on muscle mass and lean body mass in old rats. Nevertheless, we did not measure the effects of pea protein under postprandial conditions and therefore we cannot draw conclusions on the role of the leucine content on protein anabolism in old rats. Note that a second mechanisms may be involved, as we did not observe any difference between the three dietary proteins in terms of their effect on muscle protein synthesis and degradation, although we measured the rate of muscle protein turnover in postabsorptive condition. The changes observed for plantaris muscle protein synthesis in old rats were relatively in line with the changes that were observed in muscle mass. Although muscle mass tended to be higher in the whey-protein group than the pea protein group, we suggest that pea protein intake could enhance postprandial muscle protein anabolism (although we did not measure it) in old rats, which would translate into muscle protein accumulation and increased skeletal muscle mass. The influence of plant-based proteins and animal-based proteins on muscle protein synthesis has been investigated in several studies. The rate of protein synthesis in gastrocnemius muscle was lower in young rats fed raw fava bean intake than in young rats fed milk protein [41]. In addition, a lower muscle protein synthesis rate was observed in young rats when fed beans and lentils than when fed casein [34]. However, to our knowledge, the long-term effects of plant protein intake on muscle protein synthesis rate in old rats has never before been investigated.

Mitochondrial abnormalities have also been singled out as key factors in muscle changes during aging. Research on the mitochondrial electron transport chain (ETC) in skeletal muscle clearly demonstrated deficient ETC activity in muscles exhibiting the greatest loss of muscle mass with age [42]. Here, citrate synthase activity, complex 1 activity and HAD activity did not differ between dietary protein sources in old rats. Additionally, once more in old rats, we previously demonstrated that maintained mitochondrial function in skeletal muscle was associated with maintained muscle protein synthesis and muscle mass as animals aged [13]. This previous study also demonstrated that one of the mechanisms behind this action was the ability of protein intake to maintain protein turnover at the mitochondrial level [13]. This makes is tempting to postulate that pea protein, like milk proteins, could potentially help to prevent the age-related alteration of mitochondrial functional capacities in skeletal muscle, thus helping to maintain muscle mass.

### 4.4. Metabolic Parameters

We also measured metabolic parameters related to aging-related changes in muscle mass, in particular plasma pro-inflammatory and anti-inflammatory cytokine levels [43]. The increase in blood pro-inflammatory factors and the decrease in blood anti-inflammatory factors during aging causes inflammatory conditions conducive to muscle protein catabolism [44]. Here too, we showed that pea protein consumption by old rats did not modify some of the markers of the inflammatory system compared to milk proteins. It has been reported that milk protein has anti-inflammatory properties that might be effective in reducing the circulation of pro-inflammatory cytokines, such as interleukin-6 (IL-6) and tumor necrosis factor (TNF-α) [45]. A recent study on pea protein reported that a tripeptide, LRW (Leu-Arg-Trp), characterized from the pea protein legumin, and its previously studied isomer IRW (Ile-Arg-Trp) exerted strong anti-inflammatory effects by modulating the nuclear factor-κB pathway [46]. Hence, the consumption of such proteins could help keep inflammation at a level that prevents muscle protein catabolism in old rats. In addition to inflammation, insulin resistance has been described as another cause of decline in muscle protein anabolism and muscle mass in older people [47]. Here we found no between-group differences in HOMA-IR except a trend towards a reduction in insulin resistance in the PEA group compared to the CAS group. Recent studies have shown that pea glycoproteins and peptides have antidiabetic activities, in particular by reducing insulin resistance [48,49]. Therefore, it may be possible that long-term pea protein consumption could improve age-related insulin resistance in old rats. However, further studies are needed to bridge the gap between age-related inflammation and insulin resistance and pea protein intake.

## 5. Conclusions

This study, carried out in old rats, showed that, under our experimental conditions, e.g., use of protein isolates, the body uses nitrogen with the same efficiency regardless of whether it is provided by pea protein, casein or whey. This result is partly due to the high digestibility of the pea protein, together with its EAA composition, which is close to that found in milk proteins. The divergence between our results and studies using growing rats or young rats, however, has posed unresolved questions. Here, we found evidence that plant proteins would be more effective in very old animals than in young animals. Further research is warranted to find out whether this is due to an increase in the metabolic efficiency of plant proteins or a decrease in the metabolic efficiency of milk proteins with age. In addition, clinical studies should be set up to assess the quality of plant proteins in humans, in particular the elderly, taking into consideration their pathophysiological situation and their nutritional status.

## Figures and Tables

**Table 1:** Experimental diet: composition and amino acid content.

	CAS	WHEY	PEA
**Diet composition (g/100 g)**			
Protein			
*Casein*	14		
*Soluble milk protein*		14	
*Pea protein*			14
Fat (soybean oil)	6	6	6
Carbohydrates	68	68	68
Cellulose	7.5	7.5	7.5
Vitamin and mineral mix	4.5	4.5	4.5
**Calculated energy (kcal/100 g)**	**412**	**412**	**412**
			
**Amino acid content (g/100 g protein)**			
*Tryptophan*	1.17	2.09	0.87
*Threonine*	4.18	5.09	3.79
*Aspartic acid*	6.86	11.47	12.26
*Serine*	5.57	4.69	5.37
*Lysine*	7.55	9.84	7.45
*Valine*	6.16	5.23	5.25
*Proline*	10.84	4.77	4.30
*Alanine*	2.87	4.93	4.34
*Phenylalanine*	4.69	3.62	5.56
*Isoleucine*	4.79	5.26	4.67
*Glycine*	1.75	1.83	4.02
*Tyrosine*	4.19	2.76	3.28
*Arginine*	3.11	2.54	8.12
*Leucine*	8.99	12.15	8.51
*Histidine*	2.69	2.11	2.41
*Glutamic acid*	21.47	16.86	17.70
*Methionine*	2.65	2.05	1.03
*Cysteine*	0.49	2.70	1.07

**Table 2:** Composition of the protein sources.

	CASEIN Protein	WHEY Protein	PEA Protein
Protein (%)	90.2	80.9	83.6
Fat (%)	<1	4.7	<1
Carbohydrates (%)	<1	4.3	5.6
Moisture (%)	9.0	5.6	4.4
Ash (%)	<2	4.5	5.8

Compositions were obtained from technical data sheets provided by suppliers.

**Table 3:** Primer sequences used for quantitative analysis of gene expression.

Gene Name	Forward and Reverse Primers
MAFbx (Muscle atrophy F-box)	*For 5’*-AGTGAAGACCGGCTACTGTGGAA-*3’* *Rev 5’*-TTGCAAAGCTGCAGGGTGAC-*3’*
MuRF1 (Muscle RING finger-1)	*For 5’*-GTGAAGTTGCCCCCTTACAA*-3’* *Rev 5’*-TGGAGATGCAATTGCTCAGT*-3’*
HPRT (Hypoxanthine-guanine phosphoribosyltransferase)	*For 5’*-AGTTGAGAGATCATCTCCAC*-3’* *Rev 5’*-TTGCTGACCTGCTGGATTAC*-3’*

**Table 4:** Body weight, fat mass and lean mass variations over the course of the experimental study.

	CAS	WHEY	PEA
**Body weight (g)**			
Week 0	582 ± 23	577 ± 14	595 ± 28
Week 8	585 ± 22	607 ± 16	612 ± 34
Week 16	583 ± 20	605 ± 20	590 ± 39
**Fat mass (g)**			
Week 0	91 ± 8	99 ± 8	108 ± 13
Week 8	104 ± 7	129 ± 18	136 ± 21
Week 16	99 ± 18	127 ± 15	131 ± 26
**Lean Mass (g)**			
Week 0	442 ± 18	427 ± 15	434 ± 15
Week 8	431 ± 19	424 ± 14	420 ± 15
Week 16	430 ± 17	421 ± 16	403 ± 14

Week 0, week 8 and week 16 mark the beginning, the middle and the end of the experiment, respectively. Data are expressed as means ± SEM.

**Table 5:** Tissue weights in CAS, WHEY and PEA old rats after 16 weeks of different diets.

	CAS	WHEY	PEA
Plantaris (mg)	309 ± 34	300 ± 23	263 ± 0.17
Soleus (mg)	175 ± 20	173 ± 24	165 ± 13
Gastrocnemius (g)	1.52 ± 0.29	1.19 ± 0.13	1.13 ± 0.06
Quadriceps (g)	1.94 ± 0.26	1.78 ± 0.27	1.72 ± 0.24
Hindlimb muscle mass (g)	8.82 ± 0.51	8.01 ± 0.88	7.36 ± 0.61
Perirenal adipose tissue (g)	11.7 ± 2.6	15.3 ± 1.4	19.7 ± 4.6
Subcutaneous adipose tissue (g)	11.9 ± 2.3	13.3 ± 2.0	12.2 ± 2.4
Liver (g)	13.7 ± 0.9	14.3 ± 0.9	13.2 ± 1.7
Heart (g)	1.91 ± 0.05	1.88 ± 0.08	1.96 ± 0.08

Results are given as means ± SEM. Hindlimb muscle mass is the sum of plantaris, soleus, gastrocnemius, quadriceps and tibialis muscle weights.

**Table 6:** Evaluation of the protein quality of the different experimental diets during the 4-day period in metabolic cages.

	CAS	WHEY	PEA
Nitrogen intake (g)	1.47 ± 0.10	1.61 ± 0.12	1.57 ± 0.11
Fecal nitrogen (g)	0.12 ± 0.01	0.13 ± 0.01	0.14 ± 0.02
Urinary nitrogen (g)	0.86 ± 0.07	0.88 ± 0.11	0.91 ± 0.08
Nitrogen balance (g)	0.49 ± 0.08	0.60 ± 0.20	0.61 ± 0.08
Apparent digestibility (%)	91.6 ± 0.7	92.1 ± 0.7	91.8 ± 0.8
True digestibility (%)	99.9 ± 0.5	101.2 ± 0.6	100.5 ± 0.7
Net protein utilization (%)	66.3 ± 6.7	74.7 ± 6.1	81.3 ± 6.8
Biological value (%)	66.4 ± 6.9	73.8 ± 6.0	80.8 ± 6.6

Results are given as means ± SEM.

**Table 7:** Fasting metabolic parameters in plasma of old rats after the 16 weeks of different diets.

	CAS	WHEY	PEA
**Insulin sensitivity**			
Glucose (g/L)	0.955 ± 0.106	1.010 ± 0.075	0.970 ± 0.108
Insulin (ng/mM)	1.285 ± 0.585	0.678 ± 0.213	0.553 ± 0.102
HOMA-IR	6.055 ± 1.884	4.232 ± 1.392	3.202 ± 0.761
**Lipids**			
Triglycerides (g/L)	0.789 ± 0.088	0.994 ± 0.364	0.604 ± 0.176
Total cholesterol (g/L)	0.843 ± 0.073	0.878 ± 0.067	0.833 ± 0.167
**Adipokines**			
Adiponectin (µg/mL)	5.145 ± 1.240	6.355 ± 0.764	8.751 ± 1.109
Leptin (ng/mL)	4.547 ± 0.416	5.172 ± 1.170	6.730 ± 2.935
**Cytokines**			
TNFα (pg/mL)	11.93 ± 5.90	6.28 ± 2.79	11.29 ± 2.86
IL-1β (pg/mL)	155.4 ± 67.9	158.1 ± 63.9	133.5 ± 29.6
IL-10 (pg/mL)	58.56 ± 22.95	57.26 ± 22.74	54.10 ± 10.93
TNFα / IL-10 ratio	0.264 ± 0.099	0.185 ± 0.033	0.254 ± 0.071
IL-1β / IL-10 ratio	2.540 ± 0.090	2.580 ± 0.111	2.429 ± 0.049

Results are given as means ± SEM.
